# An SVM-based method for assessment of transcription factor-DNA complex models

**DOI:** 10.1186/s12859-018-2538-y

**Published:** 2018-12-21

**Authors:** Rosario I. Corona, Sanjana Sudarshan, Srinivas Aluru, Jun-tao Guo

**Affiliations:** 1 Department of Bioinformatics and Genomics, University of North Carolina at Charlotte, 9201 University City Blvd, Charlotte, NC 28223 USA; 2 School of Computational Science and Engineering, Georgia Institute of Technology, 266 Ferst Drive, Atlanta, GA 30332 USA

**Keywords:** Transcription factor, Rigid docking, Knowledge-based potential, Support vector machine, Protein-DNA binding

## Abstract

**Background:**

Atomic details of protein-DNA complexes can provide insightful information for better understanding of the function and binding specificity of DNA binding proteins. In addition to experimental methods for solving protein-DNA complex structures, protein-DNA docking can be used to predict native or near-native complex models. A docking program typically generates a large number of complex conformations and predicts the complex model(s) based on interaction energies between protein and DNA. However, the prediction accuracy is hampered by current approaches to model assessment, especially when docking simulations fail to produce any near-native models.

**Results:**

We present here a Support Vector Machine (SVM)-based approach for quality assessment of the predicted transcription factor (TF)-DNA complex models. Besides a knowledge-based protein-DNA interaction potential DDNA3, we applied several structural features that have been shown to play important roles in binding specificity between transcription factors and DNA molecules to quality assessment of complex models. To address the issue of unbalanced positive and negative cases in the training dataset, we applied hard-negative mining, an iterative training process that selects an initial training dataset by combining all of the positive cases and a random sample from the negative cases. Results show that the SVM model greatly improves prediction accuracy (84.2%) over two knowledge-based protein-DNA interaction potentials, orientation potential (60.8%) and DDNA3 (68.4%). The improvement is achieved through reducing the number of false positive predictions, especially for the hard docking cases, in which a docking algorithm fails to produce any near-native complex models.

**Conclusions:**

A learning-based SVM scoring model with structural features for specific protein-DNA binding and an atomic-level protein-DNA interaction potential DDNA3 significantly improves prediction accuracy of complex models by successfully identifying cases without near-native structural models.

**Electronic supplementary material:**

The online version of this article (10.1186/s12859-018-2538-y) contains supplementary material, which is available to authorized users.

## Background

Protein-DNA interactions play crucial roles in many cellular processes, including specific binding between transcription factors (TFs) and their DNA binding sequences in transcriptional regulation. A deeper understanding of protein-DNA interactions and their roles in TF-DNA binding specificity, gene regulatory networks and structure-based drug design requires accurate TF-DNA complex structures. However, despite technical advances in experimental structure determination, only a very small percentage (~ 3%) of structures in the Protein Data Bank (PDB) are protein-DNA complexes [1–3]. The main technical barriers in experimental structure determination, such as the difficulty in crystallizing complexes and size limitations, are not expected to be overcome anytime in the foreseeable future [4, 5]. Computational docking between protein and DNA, on the other hand, has been considered as a cost-efficient alternative to the experimental methods for filling the void in the complex structure landscape. More importantly, success in docking technology development has great potentials in structure-based, computer-aided drug design as transcription factors represent one of the prime drug targets since mutations and aberrant TF-DNA interactions are implicated in many diseases [6, 7].

Protein-DNA docking algorithms can be broadly classified into two groups, rigid docking and flexible docking [8, 9]. Rigid docking algorithms sample the relative positions between protein and DNA while keeping the conformations of both protein and DNA molecules unchanged. Flexible docking algorithms, on the other hand, also consider the conformational changes of protein and DNA when sampling different positions between protein and DNA. While the rigid docking methods are relatively simple, they are very valuable in testing the accuracy of energy functions for binding affinity and can serve as a starting point for flexible docking predictions. A number of protein-DNA docking algorithms have been developed in the past two decades [2, 10–14]. These methods generally use knowledge-based or physics-based interaction potentials, or a combination of both, to guide the docking process and select complex models. The accuracy of a docking algorithm is usually reported as the percent of cases in which the algorithm makes a good prediction in terms of root mean square deviation (RMSD), either DNA backbone RMSD or interface RMSD (iRMSD), or fraction of native contacts (NC%) between the predicted complex model and the native structure [2, 10, 15, 16].

We have previously developed two residue-level, knowledge-based TF-DNA interaction potentials for evaluation of TF-DNA binding affinity as well as for protein-DNA docking simulations [13, 16–19]. The first one is a multi-body potential, which uses DNA tri-nucleotides, called triplets, as an interaction unit of DNA to quantitate the interactions between TF and DNA molecules. This multi-body potential considers the environment of protein-DNA interactions and can capture the essential physical interactions between protein and DNA as it shows specific strong hydrogen-bond contributions at short distances as well as van der Waals repulsion and dispersion attraction [17]. The second is an orientation-dependent interaction potential that introduces an angle term to better capture the hydrogen bond interactions between protein and DNA [16]. The multi-body and orientation potentials were applied to a dataset of 38 TF-DNA complexes using a Monte Carlo-based rigid-docking algorithm [8, 16]. The docking method makes predictions by selecting a TF-DNA complex conformation with the lowest energy in each case. Docking with the orientation potential resulted in a prediction accuracy of 55% (21/38 of TF-DNA complexes) with a cutoff of 3 Å RMSD. Among the 38 test cases, five of the them with near-native structures (RMSD_nat, pred_ ≤ 3 Å) were not correctly predicted, resulting in 13% (5/38) false negative (FN) predictions. The docking program also failed to produce any near-native TF-DNA complex conformations in 32% (12/38) of the cases. Nevertheless, the docked conformation with the lowest interaction energy was predicted as a complex model in each of the 12 cases, resulting in a high number of false positive (FP) predictions. Even though DDNA3, a knowledge-based atomic-level protein-DNA interaction potential, performed better in identifying near-native protein-DNA conformations, it could not identify the cases with no near-native models [20].

Quality assessment in protein-DNA docking predictions has important implications in biological and medical applications. Docking algorithms have generally relied on interaction energy for model selection, which always predict at least one model, right or wrong [2, 10, 16, 21]. While false negative predictions may result in missed opportunities, false positive predictions represent a much bigger problem due to the enormous costs associated with drug development and tests [22, 23]. To make the docking methods more valuable and applicable to solving biological problems, a reliable confidence measure of the predicted complex models is clearly needed. Molecular dynamics (MD) simulations have been applied to filter out false positive predictions, however MD simulations are computationally expensive, especially for a large number of cases [15, 24]. Here we present a learning-based method by applying a support vector machine (SVM)-based model to evaluate the quality of TF-DNA complex models. The main features used for SVM training and testing are based on our recent study that investigates structural factors for specific protein-DNA interactions [25]. These features include protein-DNA contact area (*pdca*), the number of protein-DNA base hydrogen bonds (*pbhb*), and the number of bidentate hydrogen bonds (*bidentate HB*) between protein sidechains and DNA bases [25]. The SVM model generates a score that indicates the probability of a predicted TF-DNA complex being a native or near-native structure. Results on the testing set of 38 TF-DNA complexes show that the SVM model greatly improves prediction accuracy, from 60.8% (orientation potential) and 68.4% (DDNA3) to 84.2% (SVM). It significantly reduces the number of false positive predictions by correctly recognizing the cases that fail to generate any near-native TF-DNA complex conformations.

## Methods

### Training and testing datasets

The training dataset has 160 TF-DNA complex structures that were previously compiled for developing a knowledge-based orientation potential [16]. To generate TF-DNA complex models, the protein and DNA structures of each TF-DNA complex in the training set were separated first. Rigid-body docking simulations were then carried out with our in-house Monte Carlo-based docking program [13, 16]. A total of 400 docking simulations (200 with the orientation potential and 200 with the multi-body potential) for each protein-DNA pair in the training set were carried out in an attempt to increase the number of positive (near-native) cases for training [17]. Of the 160 TF-DNA complexes in the training set, 19 cases failed to produce all 400 models. Therefore, 141 cases that have all 400 predicted models were used for training. We used the benchmark set developed for rigid TF-DNA docking as a testing set, which has 38 non-redundant TF-DNA complex structures and no overlap with complex structures in the training set [8, 16, 26]. For each case in the testing set, 200 docking models were generated using the orientation potential. The RMSD between the predicted TF-DNA complex model and the native structure is calculated using DNA backbone heavy atoms after superimposing the protein conformations of the two complexes [10, 16]. If the RMSD is ≤3 Å, the model is labeled as a “good” (or positive) prediction; otherwise, it is a “bad” (or negative) prediction. It should be pointed out that the cutoff value for classifying a complex model as a positive or negative can be modified to a more stringent (e.g. ≤ 1 Å) or relaxed (e.g. ≤ 5 Å) value as needed for different applications. In addition, the positive or negative cases can be defined by a different metric, such as iRMSD or NC% [2, 10, 15, 16].

### Features for the SVM model

A total of four features, which include three structural features, *pdca*, *pbhb*, and *bidentate HB*, and an atomic-level protein-DNA interaction potential DDNA3, were used to train the SVM model [25–27]. Even though we found several other structural features that show clear patterns among the three specificity groups, highly specific (HS), multi-specific (MS) and non-specific (NS) in our previous study, they were not included for SVM model training since they overlap with the three selected features (data not shown) [25, 26]. The *pdca* is calculated based on the difference of solvent accessible surface area between the individual protein and DNA component and the protein-DNA complex [26]. Naccess v.2.1.1 with default parameters was used for calculating the solvent accessible surface area [28]. All hydrogen bonds between protein and DNA in a TF-DNA complex were identified with HBPLUS v.3.06 [29]. *bidentate HBs* are those that form at least two hydrogen bonds with different acceptor and donor atoms between residue and bases.

### SVM model

A non-linear SVM model with radial basis function (RBF) kernel was used for training and testing. Platt scaling was applied to transform the binary classifier into a scoring function [30]. The SVM score *p,* ranging from 0 to 1, is a probability indicating the likelihood of a protein-DNA complex to be a near-native or native structure. For the purpose of an easy comparison with other scoring methods, including the orientation and DDNA3 protein-DNA interaction potentials, we calculated *1 – p* values as the SVM score. Therefore, the lower the *1- p*, the greater the confidence in the predicted TF-DNA complex models. A linear kernel was also applied to train the SVM models to see if there are any performance differences between linear and non-linear SVM models. R package e1071, which has embedded functionalities with both linear and RBF kernels, and Platt scaling, was used for training the SVM models.

### Balanced class selection

For most of the cases in the training dataset, the number of near-native models produced from docking simulations is far lower than the number of bad models. Among all the simulation models, only 7.6% are considered positives with a 3 Å RMSD cutoff. The majority of the docked complex models (92.4%) have more than 3 Å RMSD compared to their corresponding native complex structures. To reduce training bias, we applied a technique called hard-negative mining, which applies an iterative training process by selecting an initial training dataset with all the positive cases and a random sample of the same size from the negative cases. It then trains a model based on the initial training dataset and adds to it the cases that resulted in false positives after previous training, until the training dataset remains unchanged [26].

### Performance evaluatuion

We used two methods for performance evaluation in training and testing. The first one is Matthews Correlation Coefficient (MCC), a widely-used method for assessing binary classifiers. Unlike other measures such as precision that is biased towards increasing the number of true positive cases only, MCC evaluates the performance by considering all four cases, *true positive* (TP), *true negative* (TN), *false positive* (FP), and *false negative* (FN), as shown in Eq. 1. MCC also has the advantage when the number of positive and negative cases are unbalanced, making it particularly useful for this study.

1 $$ MCC=\frac{TP\times TN- FP\times FN}{\sqrt{\left( TP+ FP\right)\left( TP+ FN\right)\left( TN+ FP\right)\left( TN+ FN\right)}} $$

In addition to MCC, prediction accuracy (Eq. 2) was used for evaluating the model performance on the testing set. A probability cut-off of 0.5 for the SVM score *p* was applied for assigning a good (*1*-*p* < 0.5) or a bad (*1*-*p* ≥ 0.5) prediction. If the best score out of 200 predictions is < 0.5 with RMSD ≤3 Å, then the case is a TP; if the best score is < 0.5, but the RMSD of the model is more than 3 Å, then the case is defined as FP; If the best score is ≥0.5, and the minimum RMSD of the 200 predictions is greater than 3 Å i.e., the docking algorithm fails to produce any good models, the case is classified as TN. However, if the best score is ≥0.5, but there is at least one near native model (RMSD ≤3 Å), then it is considered as a FN.

2 $$ Accuracy=\frac{TP+ TN}{TP+ FP+ FN+ TN} $$

## Results

**Fig. 1:**
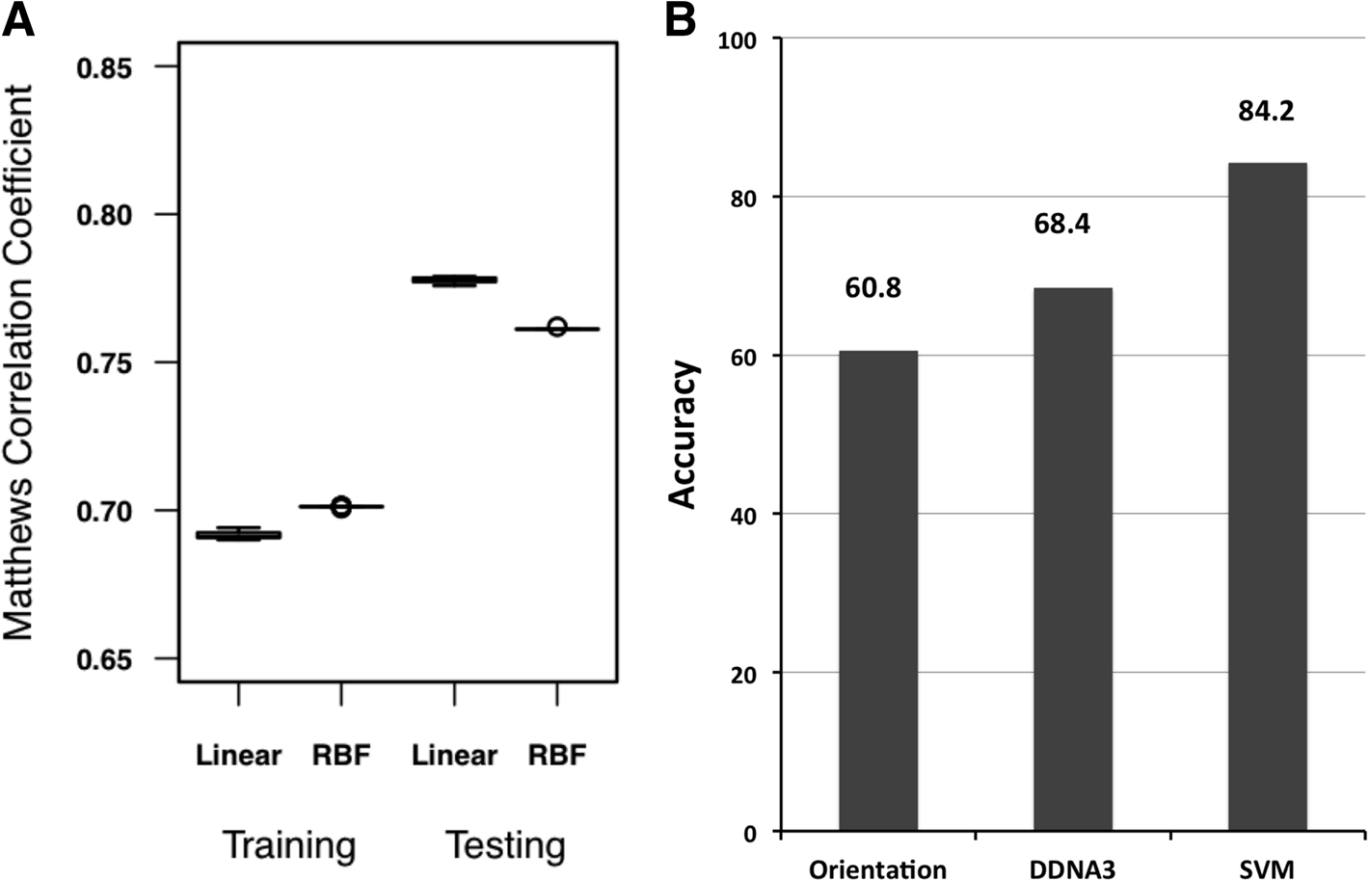
Performance of the SVM model. **a** Distribution of the Matthews correlation coefficients of 30 independent SVM models on the training and testing datasets using the linear and radial basis functions (RBF) kernels. **b** Distribution of the accuracy of the SVM model compared to the accuracy based on the orientation potential and DDNA3 potential

As described in the Methods section, hard-negative mining was applied to address the issue of unbalanced number of positive and negative models, in which an initial random sample of the negative cases was selected from the training dataset. To test the robustness of the method, 30 independent SVM models were carried out for training and testing. The overall MCC values for the training and testing datasets with linear or RBF kernels are shown in Fig. 1a. There is no apparent difference of performances between the linear kernel and the RBF kernel, 0.69 (linear) and 0.70 (RBF) for the training dataset, and 0.78 (linear) and 0.76 (RBF) for the testing set respectively. The distributions of the MCC values of the 30 independent models show very small variations, suggesting that an SVM model with the four selected features is very stable. The SVM models significantly improve the prediction accuracy 84.2% (32/38) over the orientation potential 60.8% (23/38) and DDNA3 potential 68.4% (26/38) (Fig. 1b). It is not surprising that all 30 SVM models produced the same accuracy since the models show very small variations in both the training and testing sets (Fig. 1a).

**Fig. 2:**
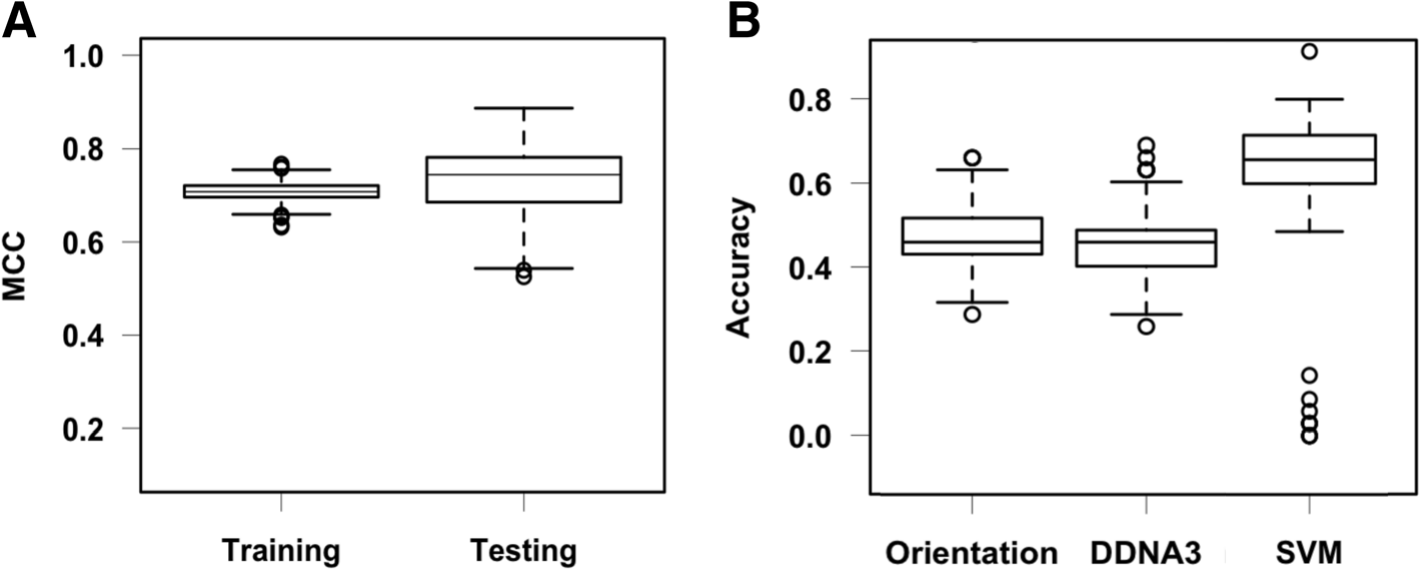
Performance of the SVM model by random splitting of the training set. The test was done by randomly splitting the original training set into training (106 complexes) and testing set (35 complexes). **a** Distribution of the Matthews correlation coefficients of 200 independent SVM models on the training and testing datasets using the radial basis functions (RBF) kernel. **b** Distribution of the accuracy of the SVM model compared to the accuracy from the orientation potential and DDNA3 potential

The MCC values of the testing set are higher than those of the training set (Fig. 1a), which may reflect the different ways of selecting protein-DNA complexes historically for the training and testing cases. For the training set, each case is a single TF chain and DNA complex [16]. However, in the testing set, the protein component in each TF-DNA complex is a transcription factor unit and DNA, which can be a single protein chain, two interacting protein chains, or even four interacting protein chains in some cases [8]. As a result, the interface area and the number of hydrogen bonds between protein and DNA in the testing set is generally larger than the cases in the training set. A larger interface area and more hydrogen bonds make it easier for accurate docking predictions [8, 31]. To test if the MCC difference is a result of the protein components between the training and testing sets, we randomly split the docking results in the training set into a new training set (106 complexes) and a new testing set (35 complexes) and repeated it 200 times. The results show that when comparable protein-DNA complexes are used for training and testing, the MCC values are similar (Fig. 2a). The testing set has a relatively larger variation than that in the training set, a possible small sample effect as the size of the testing set only has 35 cases, three times smaller than the training set. In terms of the prediction accuracy, the SVM model still outperforms both the orientation and DDNA3 potentials (Fig. 2b), though it is smaller than the benchmark testing set, which is not unexpected due to differences in the interface area and the number of hydrogen bonds (Figs. 1b and 2b).

**Fig. 3:**
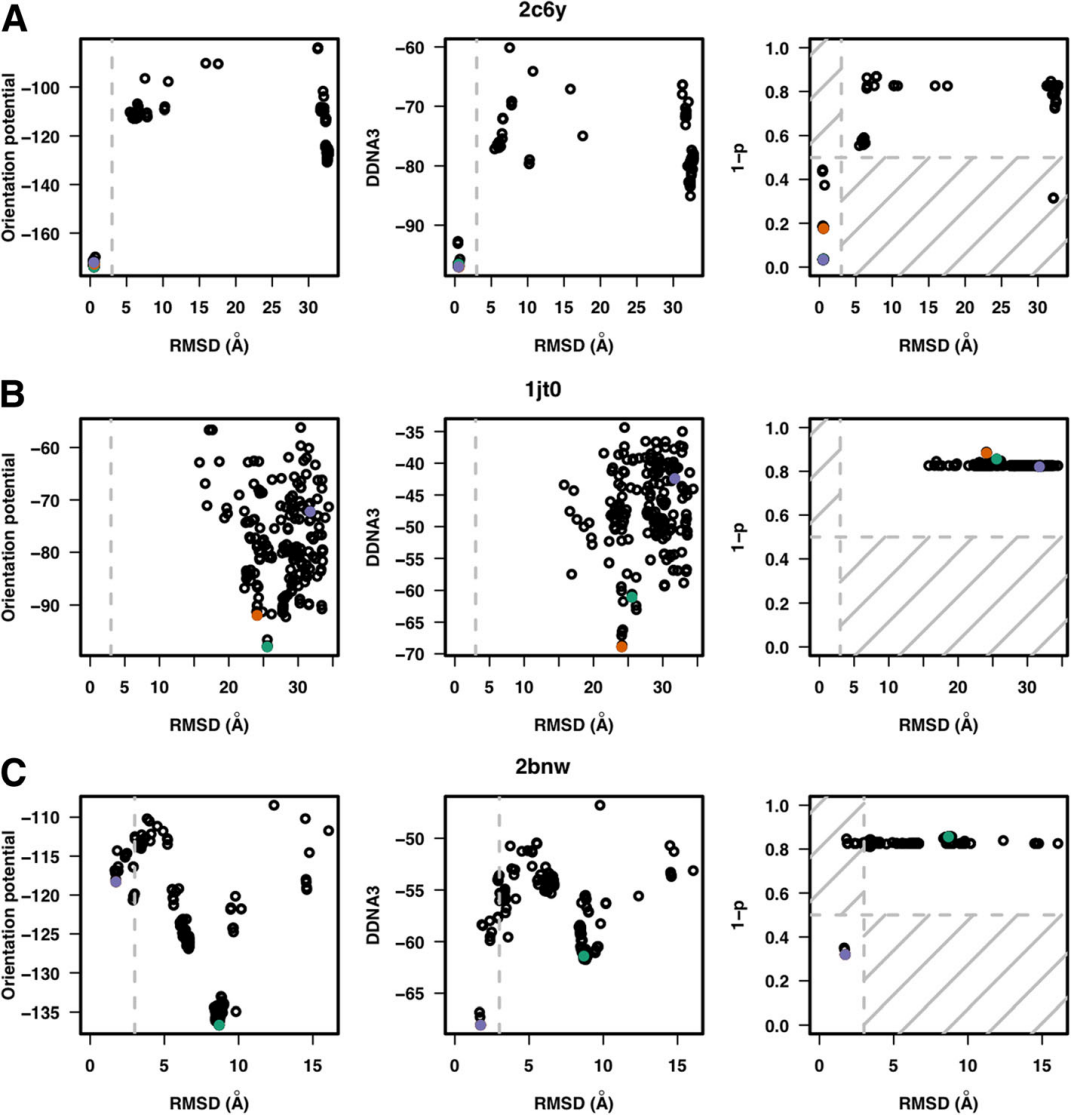
Prediction of complex models. RMSD vs. orientation potential, DDNA3 potential and SVM (RBF) quality score for three TF-DNA complexes 2c6y (**a**), 1jt0 (**b**), and 2bnw (**c**) from the testing dataset. The conformation with the lowest orientation potential (green), the lowest DDNA3 potential (orange) and the highest quality score or lowest *1- p* (blue) are highlighted across the three selection methods. The RMSD cutoff is set at 3 Å (vertical gray dashed line) and the quality score cutoff value is set at 0.5 (horizontal gray dashed line) for the SVM model

The ability to correctly predict cases that fail to produce any near-native complex models is the main contribution of the SVM model to the overall performance improvement over the interaction energy-based predictions. Ten of the 38 docking simulations (1jt0, 1rd8, 1rio, 2fio, 2ito, 2rbf, 3hdd, 1h8a, 1xpx, 1zme) did not generate any near-native complex models. The SVM model successfully identified all 10 of them while both of the interaction-based methods predicted 10 false positives. Figure 3 shows three examples that compare the predictions of the orientation potential, DDNA3 and the SVM score. Each of the three methods had a good prediction of a near-native docking structure for *2c6y* (forkhead box protein K2, FoxK2) (Fig. 3a). In the case of *1jt0* (HTH-type transcriptional regulator QacR), in which no near-native structures were generated from the docking simulations, both the orientation potential and DDNA3 selected a model with the lowest energy as the prediction, resulting in false positive predictions in both cases while the SVM model correctly predicted that there were no near-native structures produced from the docking simulations (Fig. 3b). As for *2bnw* (omega transcriptional repressor), both the DDNA3 and SVM model correctly picked one of the near-native conformations as a model while the orientation potential-based method resulted in a false positive prediction (Fig. 3c). The detailed results for all 38 cases are available at Additional file 1.

**Fig. 4:**
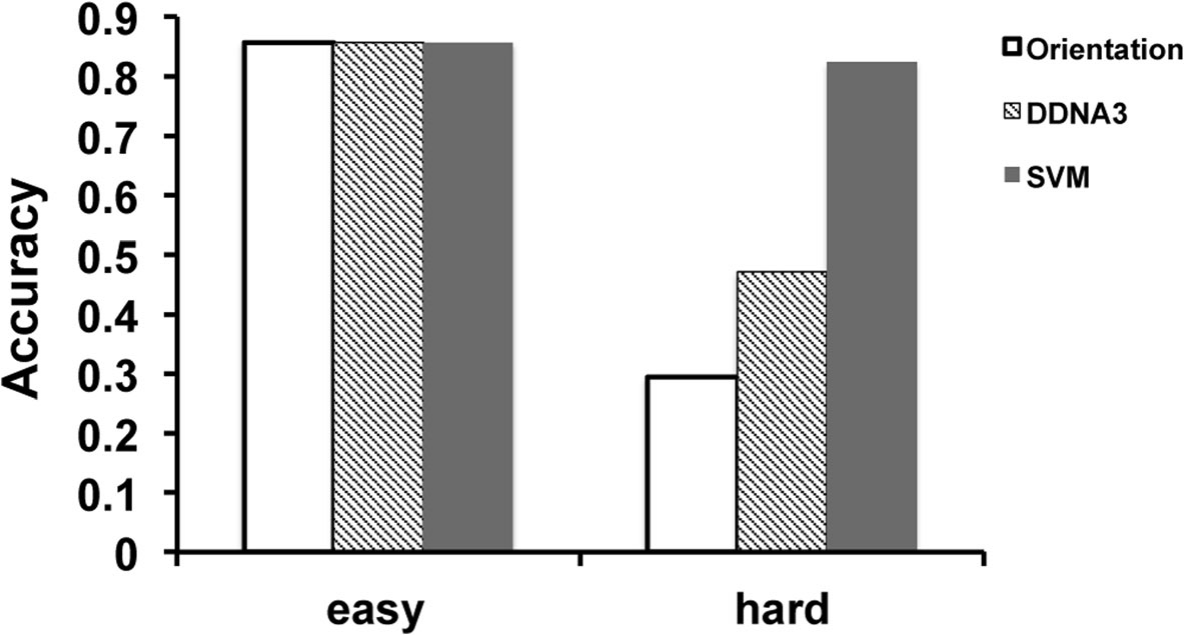
Performance of the SVM model for the easy (21 cases) and hard (17 cases) targets from rigid docking benchmark

The benchmark test set consists of 38 TF-DNA complexes that are grouped into easy and hard cases as described in our previous work [8]. The classification is based on the number of residue-base contact (NRBC). Seventeen TF-DNA complexes with fewer than 10 NRBC are classified as ‘hard’ targets and the other 21 complexes with more than 10 NRBCs are considered as ‘easy’ targets. The prediction accuracy for the easy targets is the same (18/21 = 85.7%) in all three prediction methods, 16 TP + 2 TN for SVM and 18 TP for both the orientation and DDNA3 potentials (Fig. 4). However, for the hard targets, the SVM method improves significantly over the knowledge-based orientation and DDNA3 potentials. There are 5 TP (29.4%) and 8 TP (47.1%) for the orientation potential and DDNA3 respectively while there are 6 TP + 8 TN (82.4%) predictions from the SVM model. These cases are considered as hard for docking predictions because it is very difficult to generate near-native complex models from docking simulations due to incomplete sampling and/or the lack of more accurate interaction potentials [32]. Therefore, it is critical to be able to correctly recognize the hard cases that do not have any near-native complex models.

**Fig. 5:**
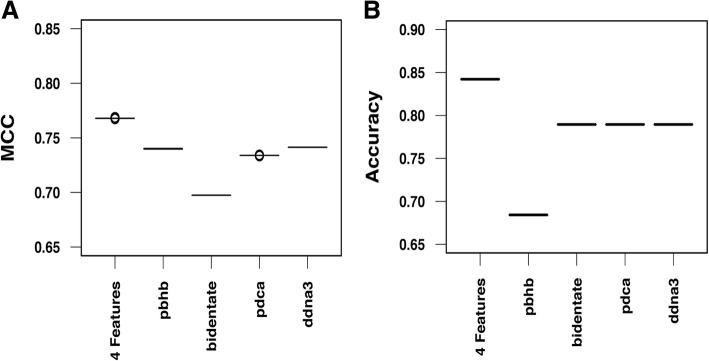
Contribution of the features to prediction performance. **a** Distribution of Matthews correlation coefficient of 30 independent SVM (RBF) models for four features, or three features by excluding *pbhb*, *bidentate* hydrogen bond, *pdca*, or DDNA3 respectively. **b** Distribution of accuracy of the SVM model for four features, or three features by excluding *pbhb*, *bidentate* hydrogen bond, *pdca*, DDNA3 respectively

The contribution of each of the four features DDNA3, *pdca*, *pbhb*, and *bidentate HB* in the SVM model to the prediction accuracy was evaluated by taking out one feature at a time and compared on the 38 test cases. Both the MCC values and prediction accuracy decreased after one of the four features is left out (Fig. 5). The MCC values and accuracy data show a slightly different trend since all the docked models are considered in MCC calculation but only one prediction from each case is used for prediction accuracy. Consistent with the results with all four features, there are very small or no MCC variations with any three of the four features. These results suggest that each of the four features contributes to the prediction accuracy to some degree and a combination of four features produces the best prediction results.

## Discussion

When a protein-DNA docking simulation generates near-native models, the knowledge-based potentials have shown success in identifying these close to native structures. However, for cases that no near-native complex conformations are produced, methods using interaction-based potentials will fail and result in false positive predictions. In this study, we developed an SVM-based model for assessing the quality of TF-DNA complex models using three structural features and DDNA3 and demonstrated that this SVM model can correctly recognize the cases without good docking solutions and reduce the number of false positive prediction significantly.

We found comparable performances in terms of MCCs and prediction accuracy between the linear and non-linear (RBF) kernels. The method is robust as there are very small MCC variations (Fig. 1) or lack of MCC variations (Fig. 5) among 30 independent SVM models, suggesting that the hard-negative mining technique can eliminate compositional bias in the training set. The SVM model and scoring scheme significantly improved the prediction accuracy over both the orientation and DDNA3 protein-DNA interaction potentials (Fig. 1). Most importantly, our SVM based scoring function, unlike the energy-based approaches, helps us correctly identify the true negatives where docking algorithms fail to produce near-native complex conformations. This is of paramount importance in applying predicted complex models in drug design as it can dramatically save time and costs if we know there are no near-native models generated from any docking program.

**Fig. 6:**
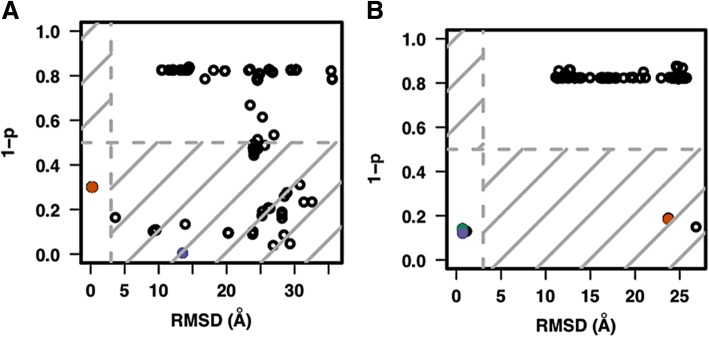
Prediction of a complex model for 2 ac0, a tetramer (**a**) and for a dimer from 2 ac0 (**b**). The conformation with the lowest orientation potential (green), the lowest DDNA3 potential (orange) and the highest quality score or lowest *1- p* (blue) are highlighted across the three selection methods. The RMSD cutoff is set at 3 Å (vertical gray dashed line) and the quality score cutoff value is set at 0.5 (horizontal gray dashed line)

While the SVM scoring model predicted much better for easy targets (~ 90% accuracy) than the hard targets in the 38 benchmark test set, we found that it failed badly for the case *2 ac0*, classified as an easy target in the rigid docking benchmark (Additional File 1) [8]. *2 ac0* is an X-ray crystal structure of p53 in complex with their target DNA sequence. Unlike other structures in the testing dataset, *2 ac0* is a tetramer (dimer of dimers) [20]. A number of docked models have good SVM scores even though they are far from the native structure (Fig. 6a). Since three structural features, *pdca*, *pbhb*, and *bidentate* hydrogen bond correlate with the size of the protein-DNA interface, we hypothesized that the extremely large contact surface between p53 and DNA and the number of hydrogen bonds may be the cause of the failed prediction. To test this idea, we separated a dimer p53 from the docked complexes and redid the SVM scoring. The SVM score correctly picked up one of the near-native conformations (Fig. 6b). Therefore, caution should be exercised when predicting TF-DNA complexes with vary large interaction surface using the SVM model.

Although the SVM model was developed with TF-DNA complex models derived from a rigid docking algorithm, the model can be applied to assess any TF-DNA complex models, either from rigid docking or flexible docking because the basic idea behind this approach relies on interactions between protein and DNA. While flexible docking is a much harder problem, our method could still be applied as a post-filter to reduce the number of false positives. In addition, depending on the need, new SVM scoring models can be trained using smaller or larger RMSD values than the one (3 Å) used in this study or using a different metric. We can envision a fully developed, efficient and accurate pipeline for TF-DNA docking predictions where the SVM model developed in this study will serve as a confidence measure for the predicted conformations or clusters of conformations.

## Conclusions

A combination of structural features that are important for specific protein-DNA interaction and a powerful learning-based SVM method can help assess the quality of complex models from docking simulations. The key contribution of the SVM model lies in its ability to dramatically lower the number of false positive predictions, which has great implications in structure-based design studies.

## Additional file

Additional file 1: Predictions of the 38 test cases using Orientation potential, DDNA3, and SVM. (PDF 595 kb)

## Abbreviations

FN: False negative
FP: False positive
HB: Hydrogen bond
MCC: Matthews correlation coefficient
MD: Molecular dynamics
NC: Native contact
NRBC: Number of residue-base contact
PDB: Protein Data Bank
RMSD: Root mean square deviation
SVM: Support vector machine
TF: Transcription factor
TN: True negative
TP: True positive
