# Supplementary material for: An SVM-based method for assessment of transcription factor-DNA complex models
Source: BMC Bioinformatics. 2018 Dec 21;19(Suppl 20):506. doi: 10.1186/s12859-018-2538-y (PMC6302363; doi:10.1186/s12859-018-2538-y)
Supplement: Supplementary file 1 — Predictions of the 38 test cases using Orientation potential, DDNA3, and SVM. (PDF 595 kb) [file 12859_2018_2538_MOESM1_ESM.pdf]

Figure S1. Predictions of the 38 test cases using Orientation potential (left), DDNA3 (middle), and SVM (right)

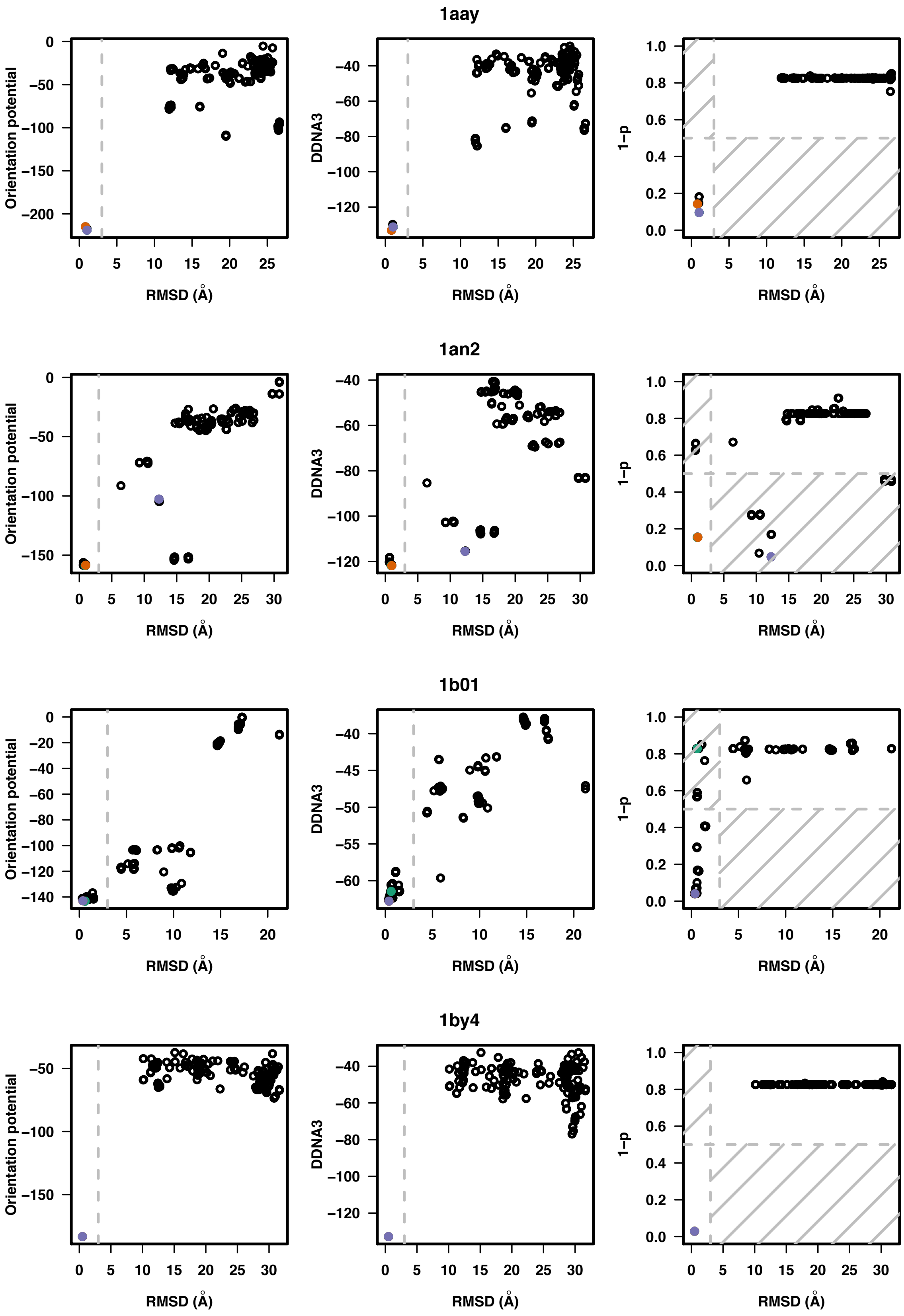
1aay
1an2
1b01
1by4
Orientation potential
DDNA3
1−p
RMSD (Å)

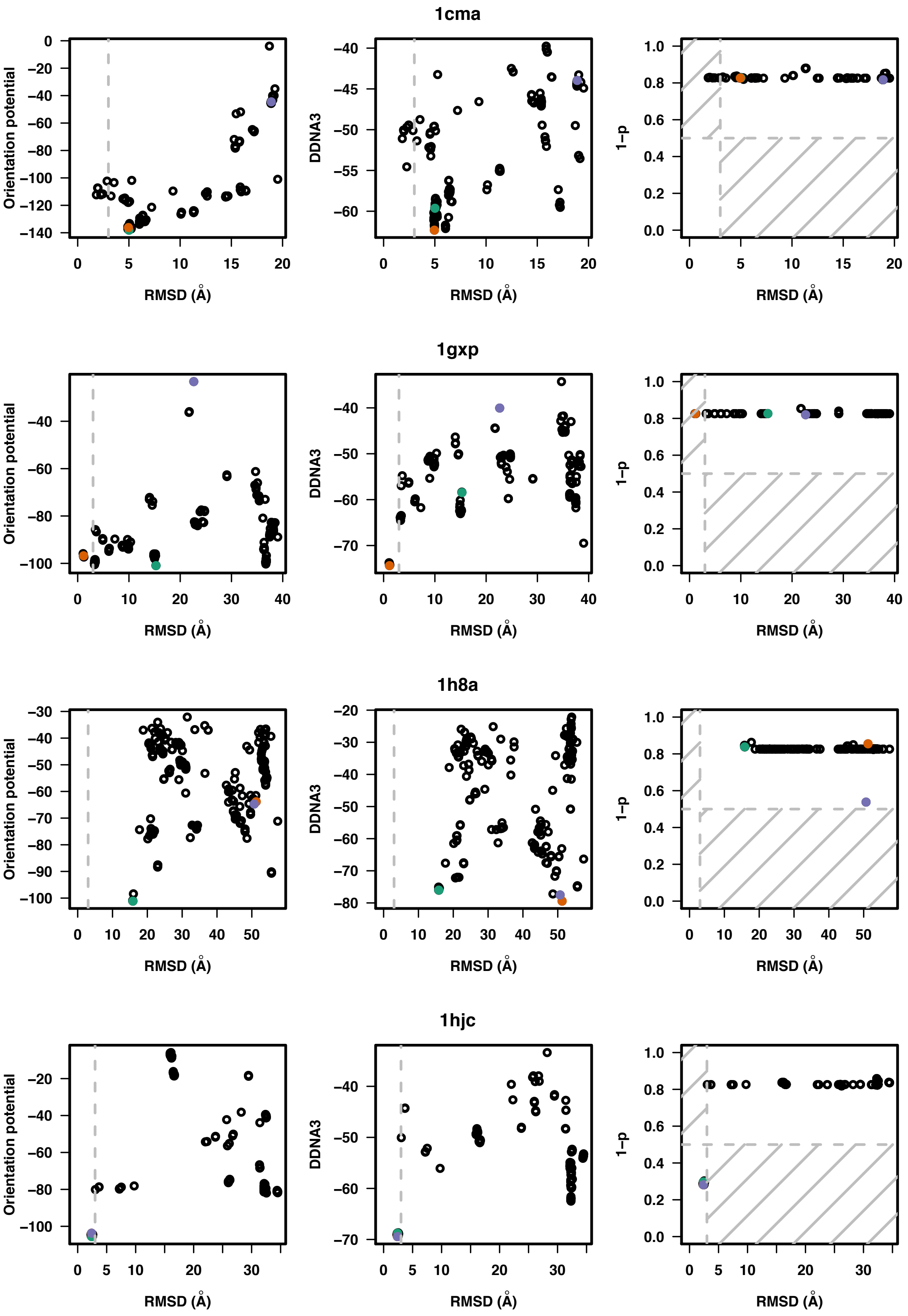
1cma
1gxp
1h8a
1hjc
Orientation potential
DDNA3
1−p
RMSD (Å)

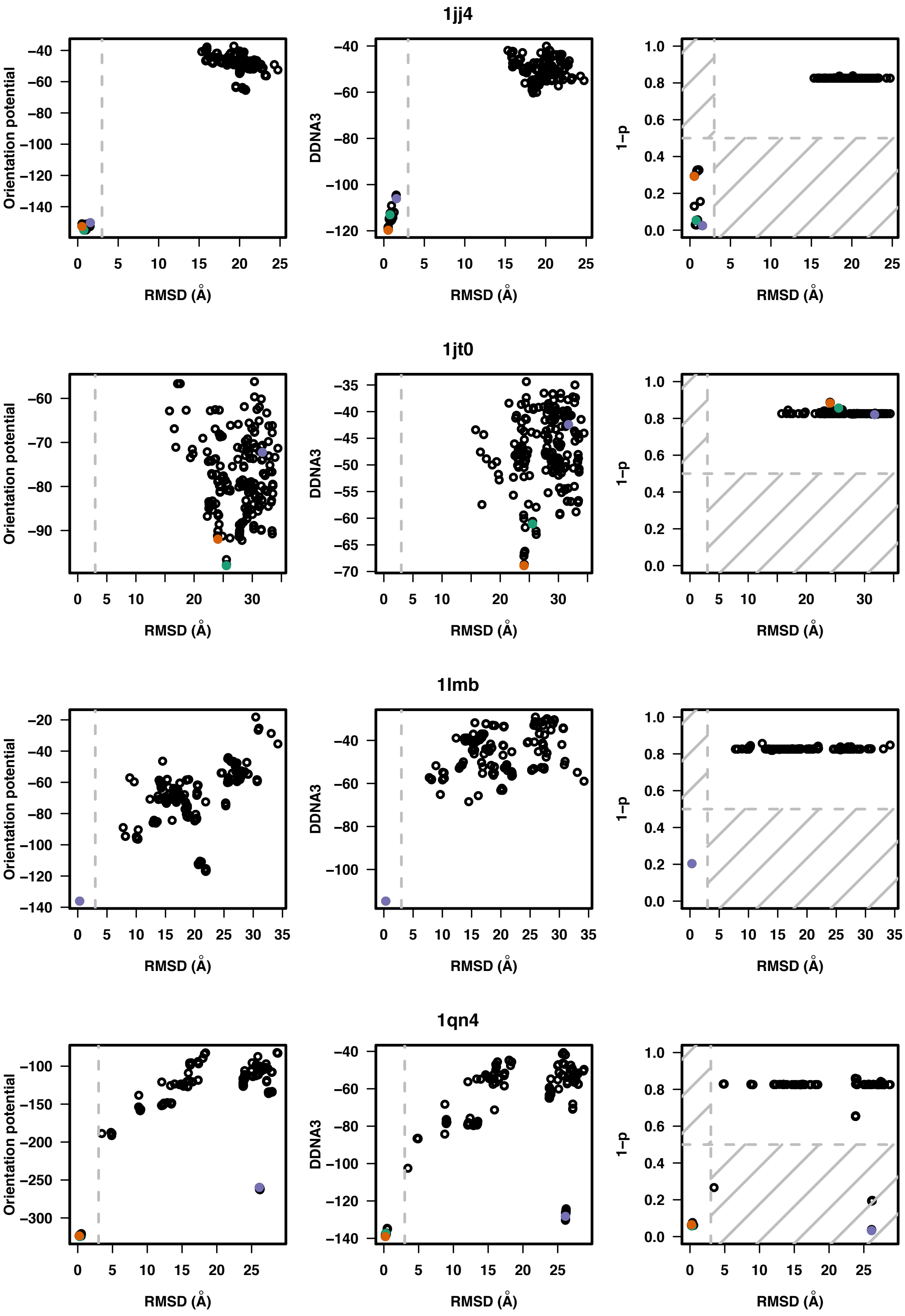
1jj4
1jt0
1lmb
1qn4
Orientation potential
DDNA3
1−p
RMSD (Å)

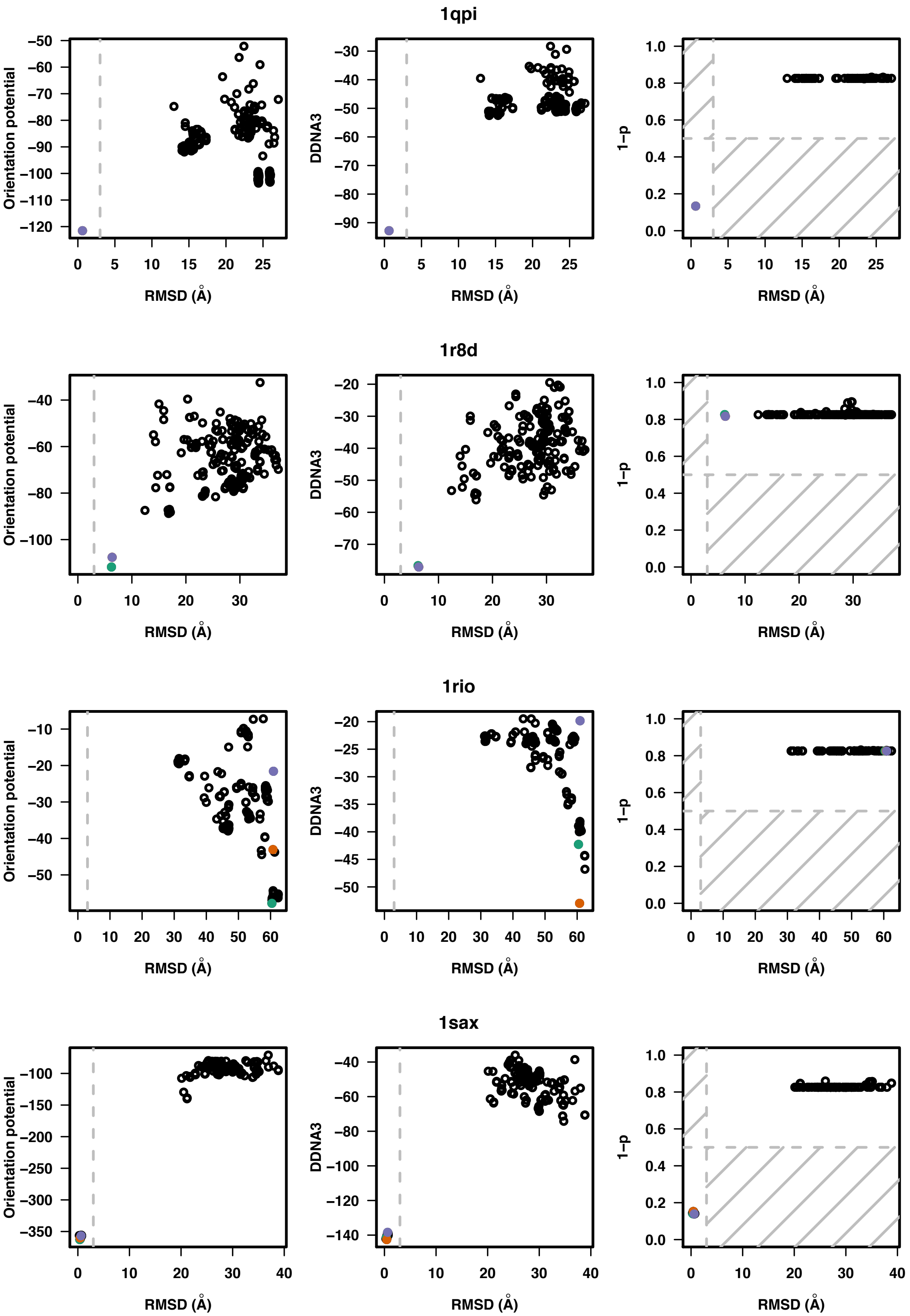

1qpi
1r8d
1rio
1sax
Orientation potential
DDNA3
1−p
RMSD (Å)

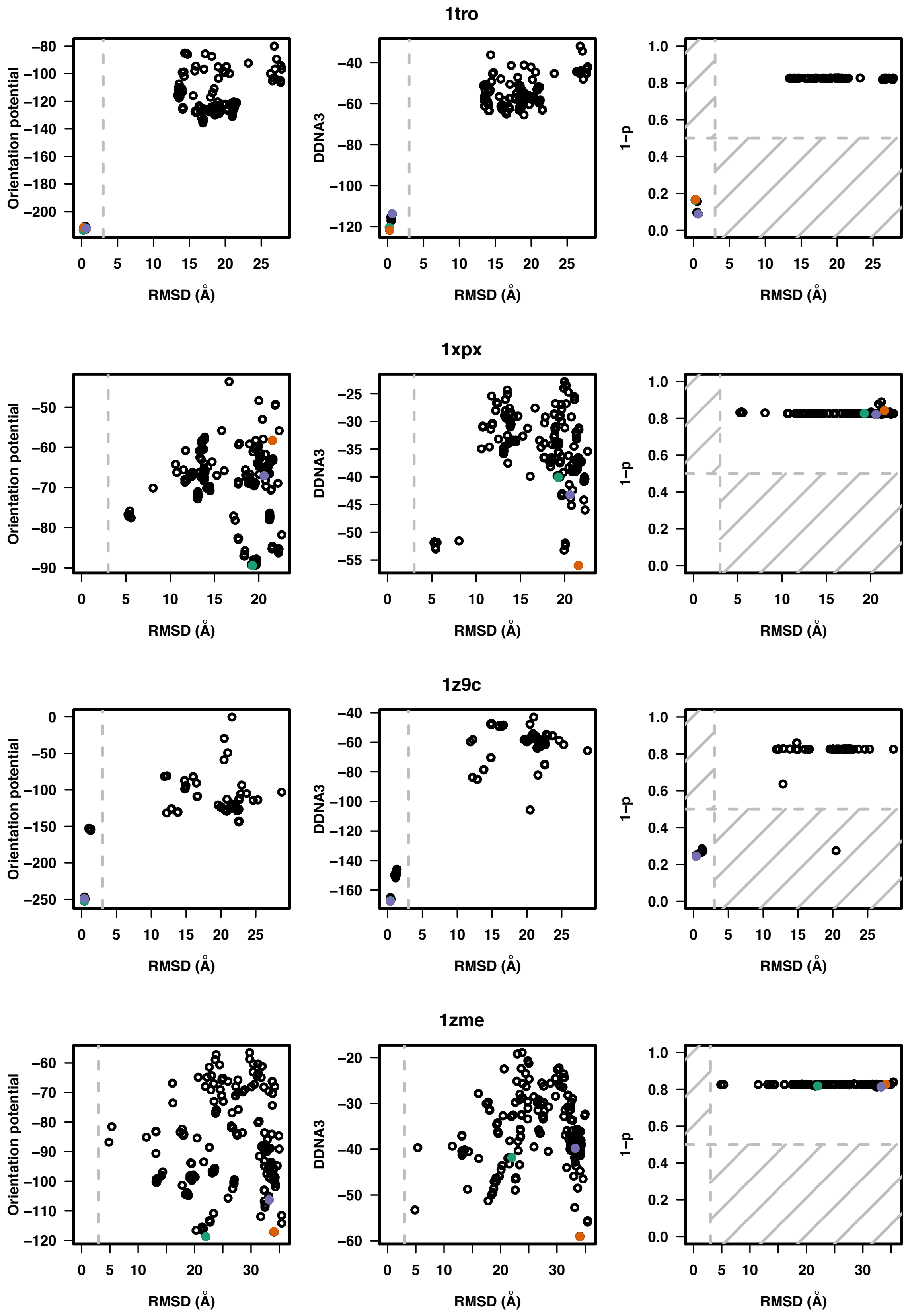

1tro
1xpx
1z9c
1zme
Orientation potential
DDNA3
1−p
RMSD (Å)

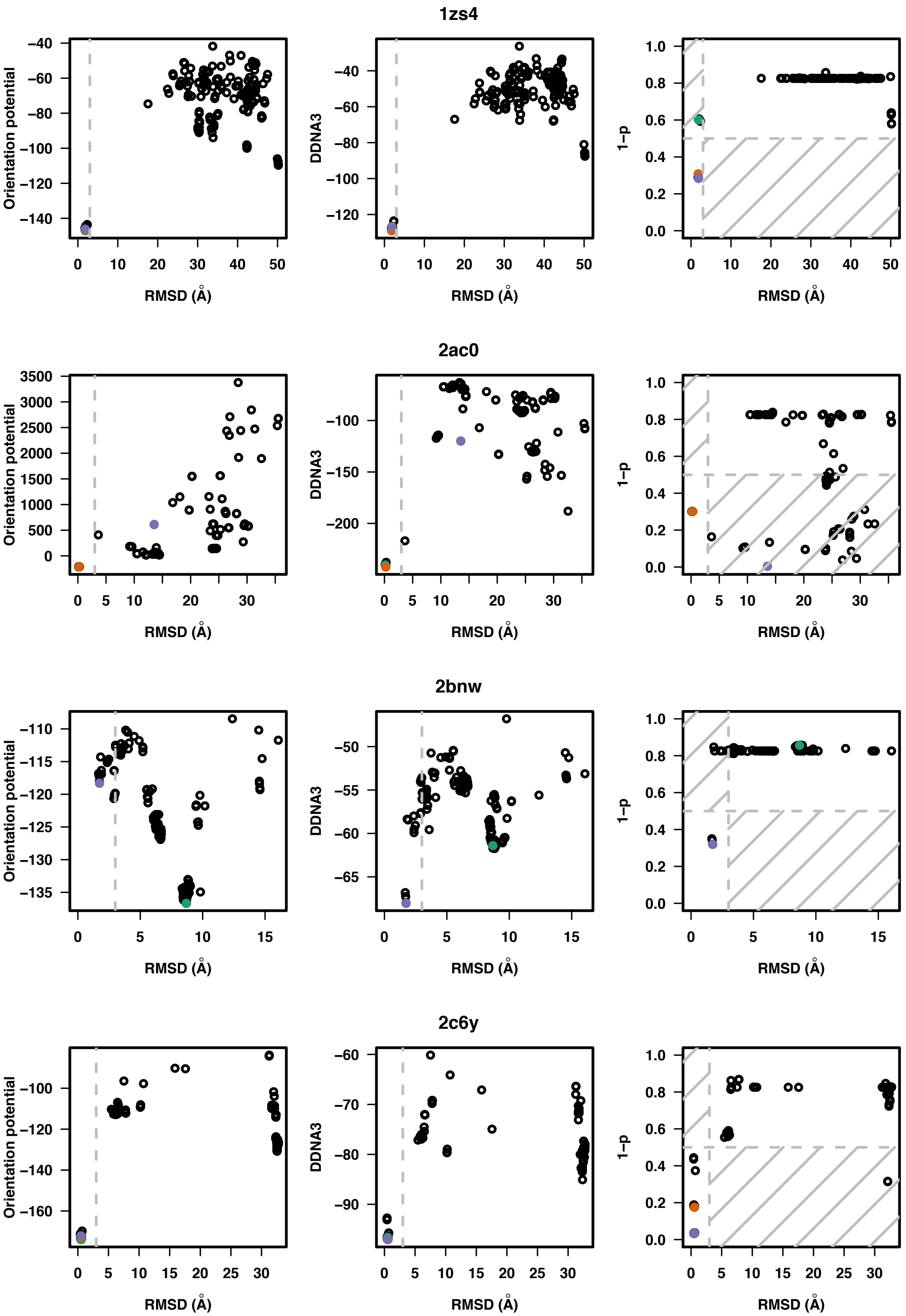
1zs4
2ac0
2bnw
2c6y
Orientation potential
DDNA3
1−p
RMSD (Å)

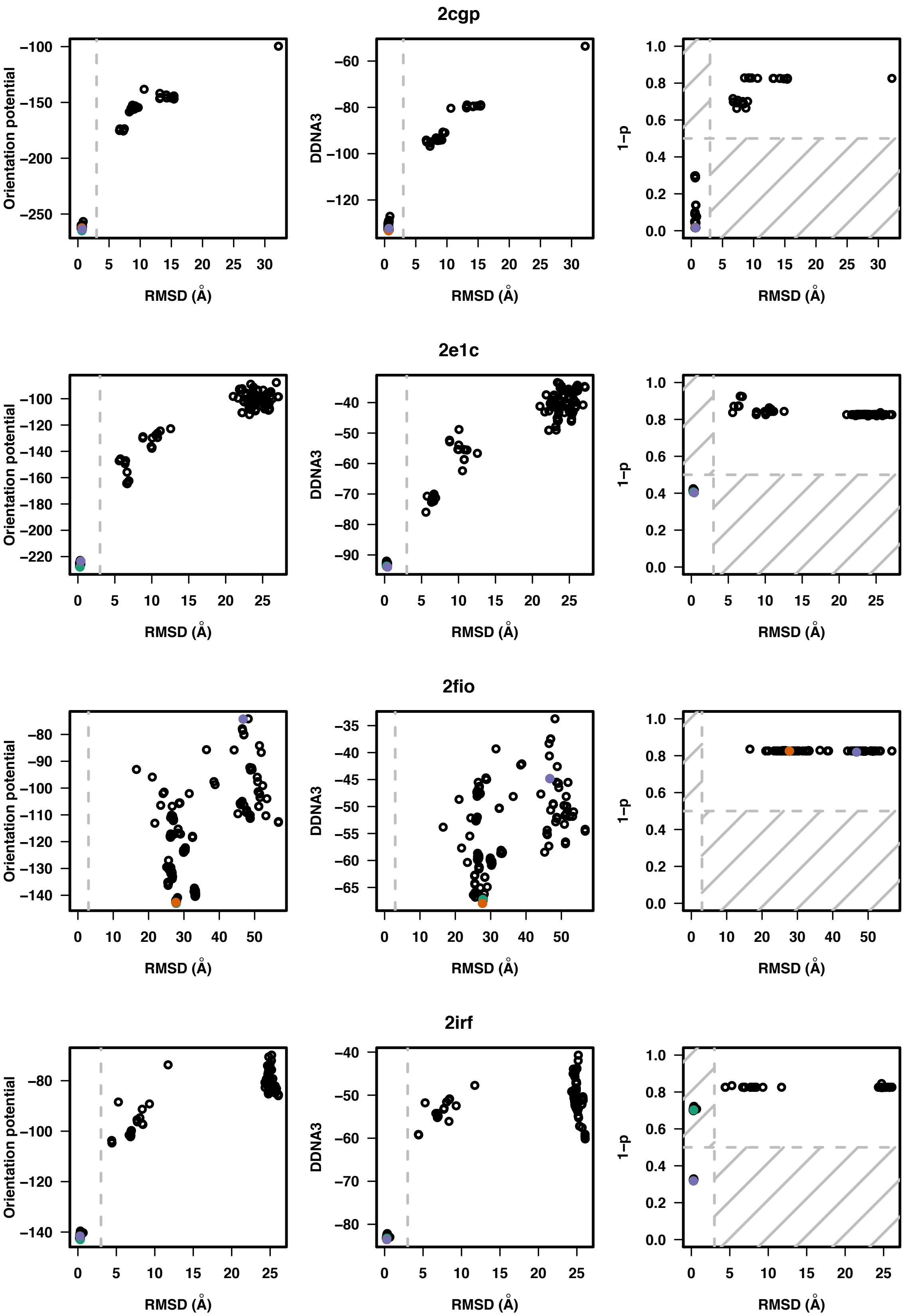

2cgp
2e1c
2fio
2irf
Orientation potential
DDNA3
1−p
RMSD (Å)

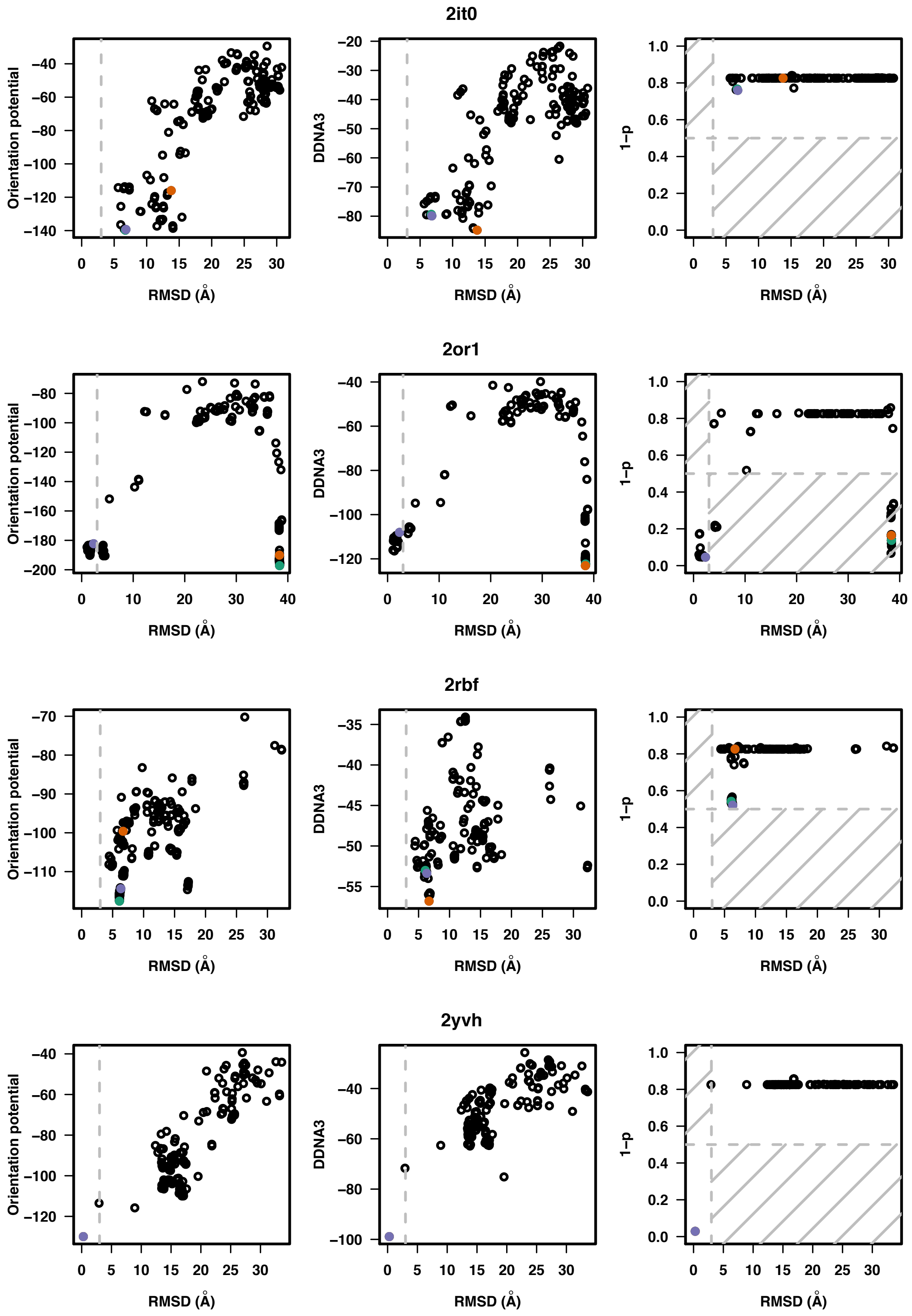
2it0
2or1
2rbf
2yvh
Orientation potential
DDNA3
1−p
RMSD (Å)

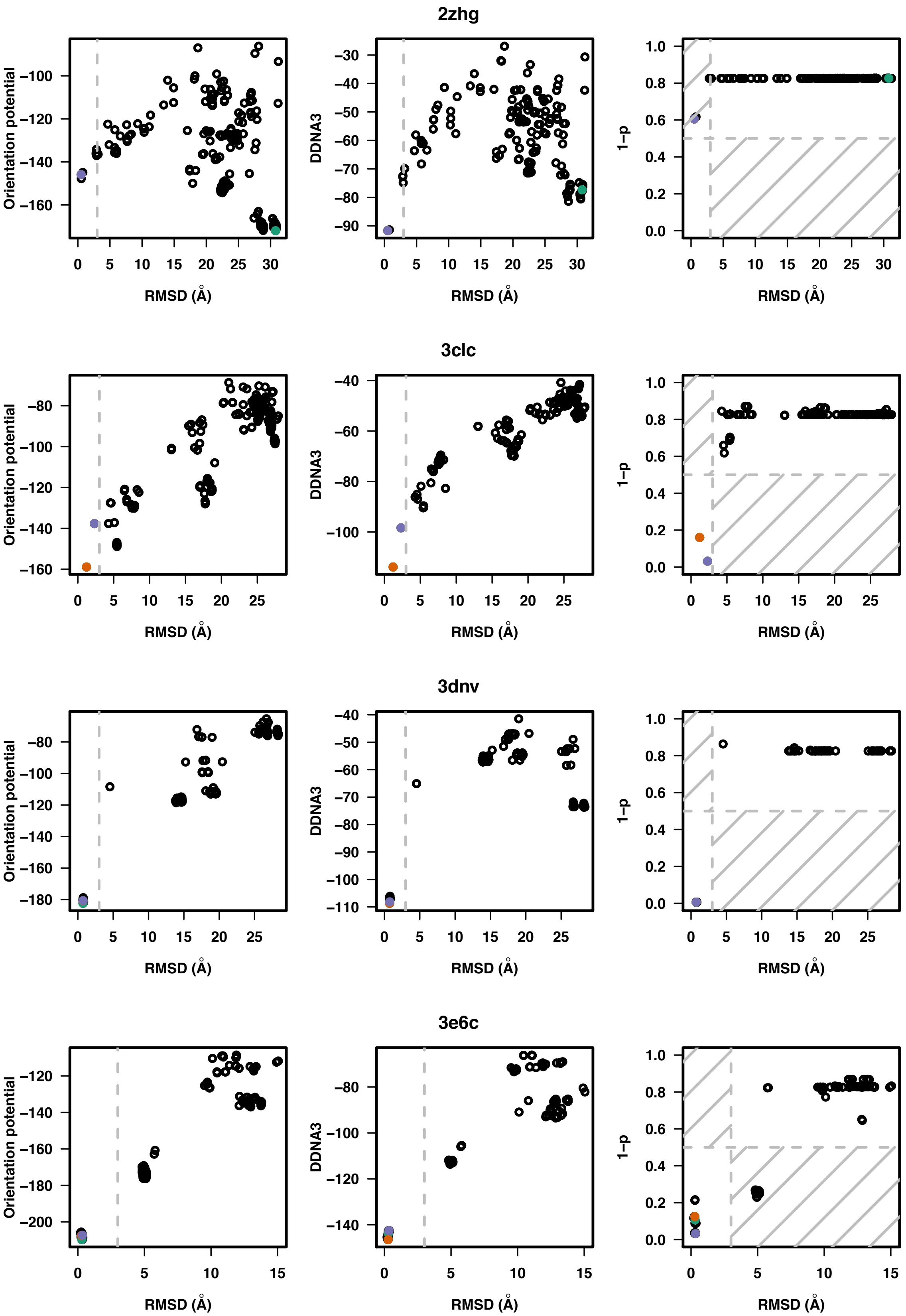
2zhg
3clc
3dnv
3e6c
Orientation potential
DDNA3
1−p
RMSD (Å)

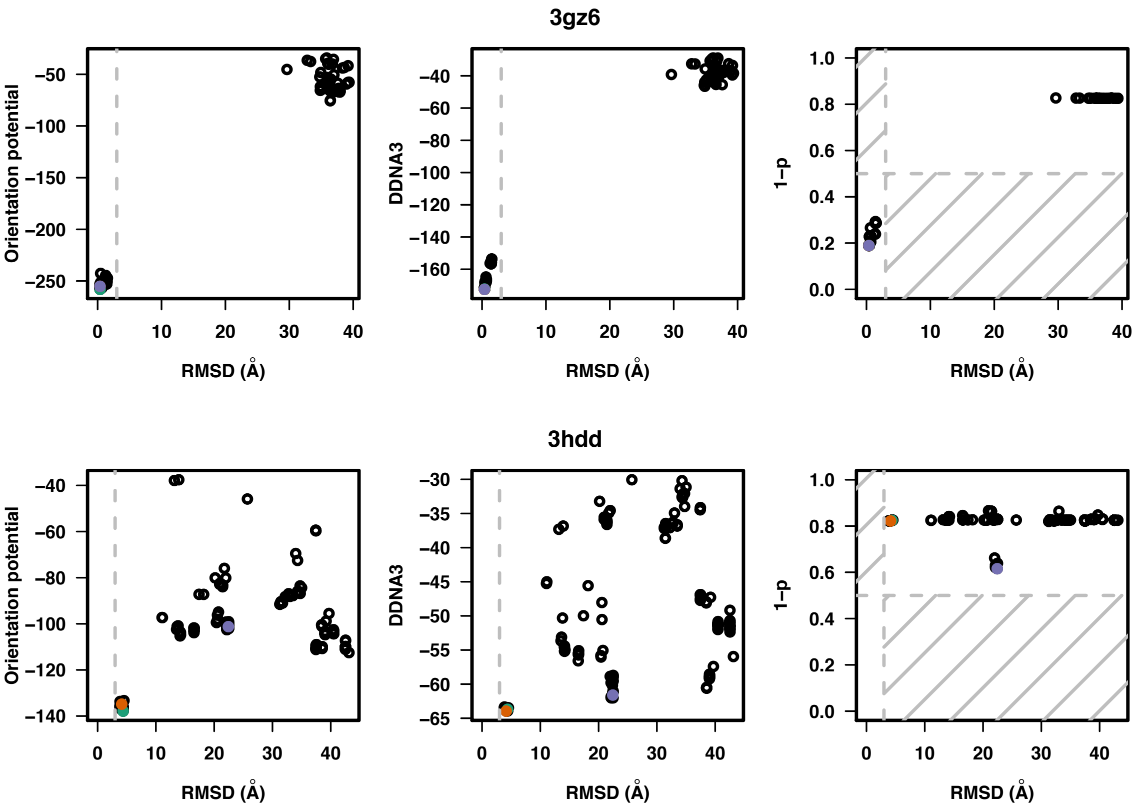
3gz6
3hdd
Orientation potential
DDNA3
1−p
RMSD (Å)
